# Electron Transfer Mechanism at the Interface of Multi‐Heme Cytochromes and Metal Oxide

**DOI:** 10.1002/advs.202302670

**Published:** 2023-08-16

**Authors:** Sheng‐Song Yu, Xin‐Yu Zhang, Shi‐Jie Yuan, Shen‐Long Jiang, Qun Zhang, Jie‐Jie Chen, Han‐Qing Yu

**Affiliations:** ^1^ Department of Environmental Science and Engineering University of Science and Technology of China Hefei 230026 China; ^2^ State Key Laboratory of Pollution Control and Resource Reuse College of Environmental Science and Engineering Tongji University Shanghai 200092 China; ^3^ Department of Chemical Physics University of Science and Technology of China Hefei 230026 China

**Keywords:** electrochemically active bacteria, electron transfer, nanomaterials, site‐directed mutagenesis

## Abstract

Electroactive microbial cells have evolved unique extracellular electron transfer to conduct the reactions via redox outer‐membrane (OM) proteins. However, the electron transfer mechanism at the interface of OM proteins and nanomaterial remains unclear. In this study, the mechanism for the electron transfer at biological/inorganic interface is investigated by integrating molecular modeling with electrochemical and spectroscopic measurements. For this purpose, a model system composed of OmcA, a typical OM protein, and the hexagonal tungsten trioxide (h‐WO_3_) with good biocompatibility is selected. The interfacial electron transfer is dependent mainly on the special molecular configuration of OmcA and the microenvironment of the solvent exposed active center. Also, the apparent electron transfer rate can be tuned by site‐directed mutagenesis at the axial ligand of the active center. Furthermore, the equilibrium state of the OmcA/h‐WO_3_ systems suggests that their attachment is attributed to the limited number of residues. The electrochemical analysis of OmcA and its variants reveals that the wild type exhibits the fastest electron transfer rate, and the transient absorption spectroscopy further shows that the axial histidine plays an important role in the interfacial electron transfer process. This study provides a useful approach to promote the site‐directed mutagenesis and nanomaterial design for bioelectrocatalytic applications.

## Graphical abstract

This work used molecular modeling and experimental measurements to explore electron transfer between bacterial cytochromes and nanomaterials. The results highlight the importance of outer‐membrane protein configuration and active center microenvironment in governing electron transfer processes. Site‐directed mutagenesis can adjust electron transfer rates, enhancing the understanding of microbe‐nanostructure interactions for improved biotic‐linked‐abiotic bioelectrocatalytic system design.

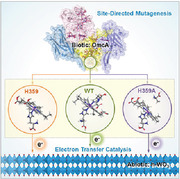

## 1 Introduction

Biotic/abiotic interfaces are widely present in various advanced technologies and natural environments, such as the microbial electrocatalysis,^[^ ^1^ ^]^ nanoscale diagnostic, and therapeutic technologies,^[^ ^2^ ^]^ human‐computer interaction,^[^ ^3^ ^]^ biogeochemical cycling of minerals.^[^ ^4^ ^]^ With the advancements in the hybrid systems comprising living organisms and nanomaterials, optimizing the biological/inorganic interface is crucial for efficient interfacial catalytic reactions.^[^ ^5^ ^]^ Specific microorganisms, termed as electrochemically active bacteria (EAB), use metal‐containing minerals as electron sinks through the EAB/minerals interface for respiration and growth.^[^ ^4^, ^6^ ^]^ In these processes, the outer‐membrane *c*‐type cytochromes (OM *c*‐Cyt) exhibit the great importance as the electron transfer catalyst for EAB to reduce the extracellular acceptors.^[^ ^7^ ^]^ However, the extracellular electrons transfer (EET) mechanism at this biological/inorganic interface involving OM *c*‐Cyts of EAB remains unrevealed yet.

These *c*‐Cyts adopt hemes composed of porphyrin rings and iron atoms as the redox active center for interfacial electron transfer catalysis.^[^ ^8^ ^]^ For instance, the tetraheme *c*‐Cyt CymA is recognized as an electron transfer protein that directly catalyzes the reduction of various pollutants using an electrode as the electron donor.^[^ ^9^ ^]^ Moreover, the decaheme MtrC, a typical OM *c*‐Cyt, can catalytically reduce H_2_O_2_ via a proton‐coupled electron transfer mechanism with a high valent iron‐oxo species as the catalytic intermediate.^[^ ^10^ ^]^ The stacked hemes serve as the active sites for the redox catalysis and act as a continuous channel for electron flow.^[^ ^11^ ^]^ To reveal catalytic mechanisms, structures of these *c*‐Cyts (e.g., OmcA, MtrC, MtrF, and OmcS) have been analyzed, revealing hemes coordinated by a pair of axial histidine (His) ligands.^[^ ^11^, ^12^ ^]^ The widespread presence of *c*‐Cyts emphasizes the indispensable role of these proteins with the active site heme in EET catalytic process. However, the effect of the amino acids of *c*‐Cyts (especially, the ubiquitous axial coordinated His residues) on electron transfer across the OM to extracellular acceptors remains unrevealed. Therefore, it is highly desired to elucidate the interfacial catalytic mechanism to optimize biotic/abiotic interfaces. In particular, site‐directed mutagenesis studies could confirm the essential role of specific residues.^[^ ^13^ ^]^ Combining molecular simulations with site‐directed mutagenesis and electrochemical measurements can provide valuable insights into the electron transfer mechanism between biomacromolecules and nanomaterials.

In this work, OmcA, known for high binding affinity to solid electron acceptors, was selected as a representative of OM *c*‐Cyts.^[^ ^12^, ^14^ ^]^ Due to the unique electronic and physicochemical properties, as well as reliable biocompatibility, hexagonal tungsten trioxide (h‐WO_3_) exhibits promising performance in various biotechnological applications, e.g., sensing biomarkers, imaging tumors, isolating EAB, and assessing their EET abilities.^[^ ^15^ ^]^ Furthermore, the redox potential of h‐WO_3_ is lower than −0.7 V (vs. Ag/AgCl),^[^ ^16^ ^]^ apparently lower than that of OM *c*‐Cyts. Thus, h‐WO_3_ was chosen as the representative solid electron acceptors. The interfacial catalytic mechanism between OmcA and the h‐WO_3_ nanomaterial was explored by integrating molecular modeling and experimental measurements. The impacts of the polypeptide chain surrounding the heme, including the axial coordinated His, on the configuration of the OmcA/h‐WO_3_ systems were examined through molecular dynamics (MD) simulations and density functional theory (DFT) calculations. Furthermore, theoretical calculation results were compared with the experimental results about the electrochemical behaviors of heme, wild type (WT) OmcA and axis‐His‐free OmcA to confirm the proposed mechanism. Interfacial electron transfer rate constants between various types of OmcA and electrodes were also estimated and compared. The interaction between these proteins and h‐WO_3_ in electron transfer was also investigated by transient absorption spectroscopy (TAS). In this way, new insights into the electrocatalytic reactions between *c*‐Cyts of EAB and h‐WO_3_ could be given, the roles of genes related to the EET of EAB could be identified and useful guidance for designing highly efficient nanodevices for bioelectrocatalysis could be provided.

## 2 Results and Discussion

### 2.1 Electron Transfer Catalytic Reaction between OM *c*‐Cyts and h‐WO_3_ Nanomaterial

As described by Faughnan coloration model,^[^ ^17^ ^]^ the reduction of h‐WO_3_ is attributed to the simultaneous acceptance of electrons and cations into the tunnel structures on (001) surface of h‐WO_3_ via the electron transfer catalysis by OmcA.^[^ ^18^ ^]^

**Figure 1:**
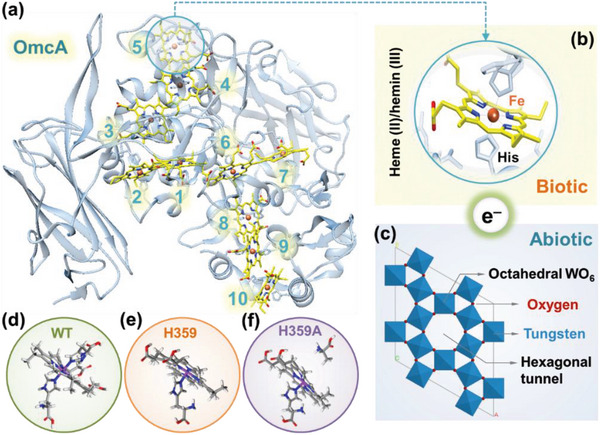
OmcA configuration and the h‐WO_3_ cell structures. a) Stereoview of molecular configuration of *Shewanella oneidensis* MR‐1′s OmcA from PDB (4LMH). b) Partially enlarged details of bis‐His‐coordinated porphyrin plane (heme 5). c) 4 × 4 × 1 supercell of h‐WO_3_ in polyhedral representation: *a* = *b* = 29.1928 Å, *c* = 3.8992 Å, *α* = *β* = 90°, *γ* = 120°. d) Bis‐His‐coordinated heme 5 in the OmcA of the WT. e) One axial His (H359) removed in the site‐directed mutagenesis. f) H359 replaced by alanine (H359A).

In the electron transfer reaction, the active center of the OmcA (PDB ID: 4LMH) can carry one electron through the reduction of Fe(III) to Fe(II) (**Figure** **1**a). Electrons are assumed to be transferred from Fe(II) in the reduced heme of *c*‐Cyt to W(VI) in oxygen‐terminated surface (001) of the h‐WO_3_. The partially enlarged part shows the details of bis‐His axial ligands bound to porphyrin plane at Fe site (Figure 1b). Subsequently, the hexagonal tunnels in the framework of a vertex‐shared WO_6_ octahedral (Figure 1c) act as the intercalation hosts for the simultaneous capture of cations and electrons from tungsten bronzes MWO_3_. The cations originate from the culture medium, with sodium ion (Na^+^) being the most abundant species in our experiments.^[^ ^15a^ ^]^

Hemes, the redox active centers of *c*‐Cyt in reduction state, are the macro‐heterocycles with iron (Fe) atom at the center and four pyrrole sub‐ligands. Along the charge carrier heme chains of OmcA, heme 5, exposed to the protein surface, is reported to be the active site of the electron transfer catalysis between *c*‐Cyt and the extracellular electron acceptor.^[^ ^19^ ^]^ Also, heme 5 shares similar coordination environments with heme 10.^[^ ^12a^ ^]^ Considering the important role of axial His in the electron transfer catalysis,^[^ ^10^ ^]^ heme 5 with an axial ligand His 359 was selected as the mutation site to construct two OmcA variants. To reveal the role of His, the His 359 coordinated to heme 5 was removed (H359, Figure 1e) and replaced by alanine (H359A, Figure 1f) as the comparative systems.

**Figure 2:**
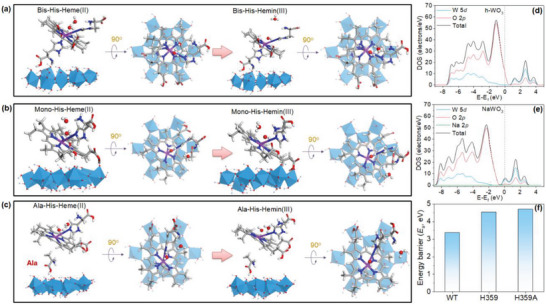
Optimized structures, electronic structure, and the kinetic properties of the electrocatalytic systems. a) Bis‐His‐Heme/h‐WO_3_ system including H_3_O^+^ and H_2_O for proton coupled electron transfer reaction in front view and top view. b) Mono‐His‐Heme/h‐WO_3_ system. c) Ala‐His‐Heme/h‐WO_3_ system. The detailed reactions are shown in Table S2 (Supporting Information). d) PDOS and TDOS plots of W and O atoms in h‐WO_3_. e) PDOS and TDOS plots of W, O, and Na atoms in NaWO_3_. f) Energy barrier of the interfacial electron transfer catalytic reactions for (a–c) systems.

The geometry structure of the aromatic porphyrin structure may be altered by the site‐directed mutagenesis, which could further impact the electron capturing by the h‐WO_3_. Table S1 (Supporting Information) presents a comparison of the structural parameters before and after the electrocatalytic reaction in three heme‐WO_3_ complexes (**Figure** **2**a–c). The lengths of the Fe‐N bond (Table S1, Supporting Information) of the three systems remain relatively consistent in both the oxidized (hemin) and reduced states (heme). However, the bond angles of N‐Fe‐N deviate significantly from 180°. These findings suggest that, upon the disappearance of one axial His ligand, the highly conjugated structure of the porphyrin ring becomes distorted, thereby decreasing the N─Fe─N bond angle.

The possible orientations of the porphyrin ring concerning the h‐WO_3_ surface for the electron capturing process were considered. For mono‐His‐Heme, the model with the heme adopting a 45° orientation exhibited a more negative adsorption energy (Δ*E*_ad_) (Figure S1, Supporting Information) compared to the paralleled orientation of heme to the h‐WO_3_ surface, suggesting that the 45° orientation was more stable. As a result, in the energy‐minimized structures of mono‐His‐Heme and mono‐His‐Hemin, the porphyrin plane also formed a 45° angle with the h‐WO_3_ crystal surface (Figure 2). For bis‐His‐Heme of WT OmcA, the steric effect of the two axial His residues led to the orientation of porphyrin ring at a 45° angle relative to the crystal surface. In such an orientation, the distance between the redox centers can be reduced to accelerate the electron transfer (Figure S2, Supporting Information), as the electrocatalytic reactions are highly distance‐dependent.^[^ ^20^ ^]^ Thus, the two axial His residues might influence the electron transfer rate. The axial ligands also play an important role in stabilizing the metal center to maintain or enhance the Fe(II)/Fe(III) redox cycling in natural environments.^[^ ^21^ ^]^

In addition, to explore the electronic structure of h‐WO_3_ and the role of Na^+^ intercalation, the densities of the states for the h‐WO_3_ and the sodium‐tungsten bronze were calculated and are presented in Figure 2d,e. After capturing bioelectrons from the active center of OmcA, the electronic structure of the nanocluster changed. The zero point on the energy axis of the plots corresponded to the Fermi level of the systems. The partial density of states (PDOS) plots of the relevant atoms can be used to further examine the main contributions to the valence and conduction bands. The mixing of W 5*d* and O 2*p* yielded the bonding states that belong to the valence band, and the antibonding states that contribute to the conduction band. On the low‐energy side, a large contribution to the density of states was from the Na 2*p*‐orbital, a feature not seen in the present work. With the injection of Na^+^, the total density of state (TDOS) and PDOS moved to the low‐energy side, and the relative Fermi level was shifted into the conduction band. The electronic structure of the h‐WO_3_ nanocluster near the Fermi level was inevitably influenced by the intercalation of Na^+^ and electrons from hemes.

### 2.2 Energy Change and Kinetics of the Electron Transfer Catalysis

To gain insight into the energy change of EET from heme(II) to the h‐WO_3_ nanostructure, DFT calculations were performed under the standard conditions and a positive free energy change (Δ*G*) value was obtained (Table S2, Supporting Information). Moreover, the measured reduction potential of h‐WO_3_ (<−0.7 V vs. Ag/AgCl) was more negative than that of OM *c*‐Cyt.^[^ ^16^ ^]^ These results indicate that extra energy was required in the electron transfer from porphyrin to h‐WO_3_. Besides, such a negative potential of h‐WO_3_ also implies that the interfacial coupling played an important role in the electron transfer catalysis. The electron transfer from porphyrin to h‐WO_3_ was the oxidation process for the porphyrin. The required external energy (≈5.2 eV) was almost the same, indicating that the mutation of one axial His residue on the active sites had a slight impact on the thermodynamic properties of electron transfer to the nanocluster. Additionally, the experimental reduction potentials (*E*_red_^⊖^) of −0.32 to −0.10 V for OM *c*‐Cyt of *Shewanella* (vs. normal hydrogen electrode)^[^ ^22^ ^]^ via the interaction with insoluble Fe(III) substrates indicate that the oxidation of OM *c*‐Cyt was not thermodynamically spontaneous. This could be explained by *E*_red_^⊖^ of the half‐reaction. The details are described in the section of Test S2 (Supporting Information). The reported experimental *E*_red_^⊖^ of the OM cytochrome reveals that the Δ*G*_red_^⊖^ was negative. Thus, the reverse reaction, the Δ*G*_ox_^⊖^ was positive. The calculated results from these systems shown in Figure 2a–c are in good agreement with the experimental results.^[^ ^22^ ^]^ These models were appropriate to describe the interfacial electron transfer process from the active center of OM cytochrome to the h‐WO_3_.

The electron transfer rate also has a great impact on the electron‐accepting performance of the h‐WO_3_. The kinetic analysis shows that there was an energy barrier for each system in the electron transfer from heme(II) to the h‐WO_3_ (Figure 2f). The rate constants can be calculated from the following equation:

(1) $$\;k_{\mathrm{et}}\;=\;\;A\exp\;\;\left(-\frac{E_{a}}{RT}\right)\;=\;\frac{k_{B}T}{h}\;e{\left(c^{⊖}\right)}^{1-n}\exp\;\left(\;\frac{\Delta^{\neq }S^{⊖}}{R}\;\right)\;\exp\;\left(\;-\frac{E_{a}}{RT}\right)$$

where *k*_et_ is the rate coefficient of electron transfer (mol^1‐^ *^n^*/L^1‐^ *^n^* ·s, for order *n*), *A* is the pre‐exponential factor (mol^1‐^ *^n^*/L^1‐^ *^n^* ·s), *E_a_* is the energy barrier (eV), *k*_B_ is Boltzmann constant (1.381 × 10^−23^ m^2^ kg·s^−2^·K^−1^), *h* is Planck constant (6.626 × 10^−34^ m^2^·kg·s^−1^), *c* ^⊖^ is the standard molar concentration (1 mol·L^−1^), *n* is the order of the reaction, and Δ^≠^ *S* ^⊖^ is the entropy of activation (J·mol^−1^·K ^−1^).

The presence of two axial His residues in the cytochrome might influence the electron‐transfer rate because of the largest steric effect, but the *E*_a_ value was the lowest (Table S3, Supporting Information). After one axial His residue was removed or replaced, the electron transfer rate from porphyrin ring to the h‐WO_3_ surface decreased. Combined thermodynamic and kinetic analysis shows that the electrocatalytic reaction can occur with a thermodynamic driving force generated by EAB during their substrate metabolism.^[^ ^23^ ^]^ This driving force enables the reaction to reach equilibrium with a tunable rate that can be adjusted by site‐directed mutagenesis. This finding provides a valuable strategy for redistributing electron flow and artificially choosing the appropriate electron transfer rate according to various electron acceptors.

### 2.3 OmcA/h‐WO_3_ Configurations

**Figure 3:**
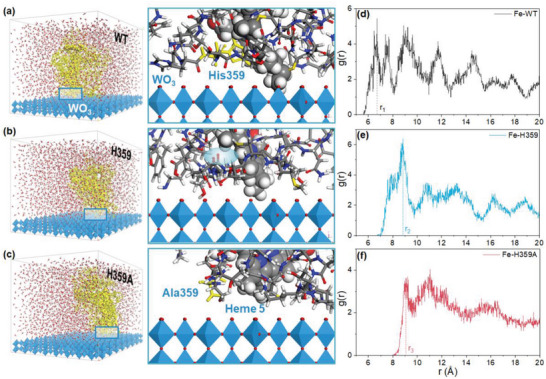
Microstructural features of the OmcA and its variants on the h‐WO_3_ (001) surface. a–c) Solvated WT/h‐WO_3_, H359/h‐WO_3_, and H359A/h‐WO_3_ systems after equilibrium, the polypeptide chain is highlighted. Zoom in at the interface between protein and crystal surface of h‐WO_3_ in the solvated systems shown in (a‐c). d–f) RDFs for the Fe atom of the heme in contact with the surface and the top most W atoms of the h‐WO_3_ slab in WT/h‐WO_3_, H359/h‐WO_3_, and H359A/h‐WO_3_.

The OmcA/h‐WO_3_ and the variant systems were relaxed by MD simulations under standard conditions (0.1 MPa, 298 K). MD simulations were performed to find out the optimal configurations for OmcA (WT, H359, and H359A) on the h‐WO_3_ surface. Initially, the protein molecule, specifically domain II including heme 5, was positioned with its mass center 30 Å above the h‐WO_3_ surface in a simulation box. Heme 5, which is ligated to His‐359, −368 at the protein/crystal interface, plays a crucial role in influencing the electron transfer rate. Following the MD simulations (Figure S3, Supporting Information), the equilibrium states reveal that OmcA and its variants approach to the crystal surface of the h‐WO_3_ at an angle of ≈45°, positioning heme 5 as the most efficient site for electron delivery (**Figure** **3**). Such an orientation of porphyrin ring is consistent with the findings from DFT calculations (Figure 2a–c) for both the oxidized and reduced states. The agreement between the MD simulations and DFT calculations highlights the significance of the 45° orientation in facilitating efficient electron transfer between OmcA and the h‐WO_3_ surface.

The radial distribution functions (RDFs, Figure 3d–f) provide insight into the distance between the Fe atom of heme 5 in contact with the surface and the top W atoms of the h‐WO_3_ at the maximum probability. In the WT/h‐WO_3_ system, the highest peak (r_1_) is observed at 6.7 Å, which is shorter than the distance r_2_ and r_3_ for the H359/h‐WO_3_ and H359A/h‐WO_3_, respectively. This result suggests that the distance between the solvent‐exposed heme and the crystal surface can be expanded by the mutation of the axial His, with the peaks of high g(r) in the WT/h‐WO_3_ system appearing at a shorter distance range (Figure 3d). The geometric structures at equilibrium state also indicate that the heme 5 was in contact with the h‐WO_3_ in the WT/h‐WO_3_, while a distance of ≈9 Å with the highest probability still exists in the H359/h‐WO_3_ and H359A/h‐WO_3_ (Figure S4, Supporting Information). In addition, the RDFs provide valuable information about the number of electron‐accepting sites available at the crystal surface, as indicated by the g(r) curve. In the WT/h‐WO_3_ system, a relatively larger number of sites are available for electron transfer compared to the other variant systems. Consequently, the distance and sites of electron transfer between protein and inorganic nanomaterials could be modulated through site‐directed mutagenesis, allowing for greater control over the bioelectrocatalytic process.

### 2.4 Electrochemical Behaviors of OmcA and its Variants on h‐WO_3_

**Figure 4:**
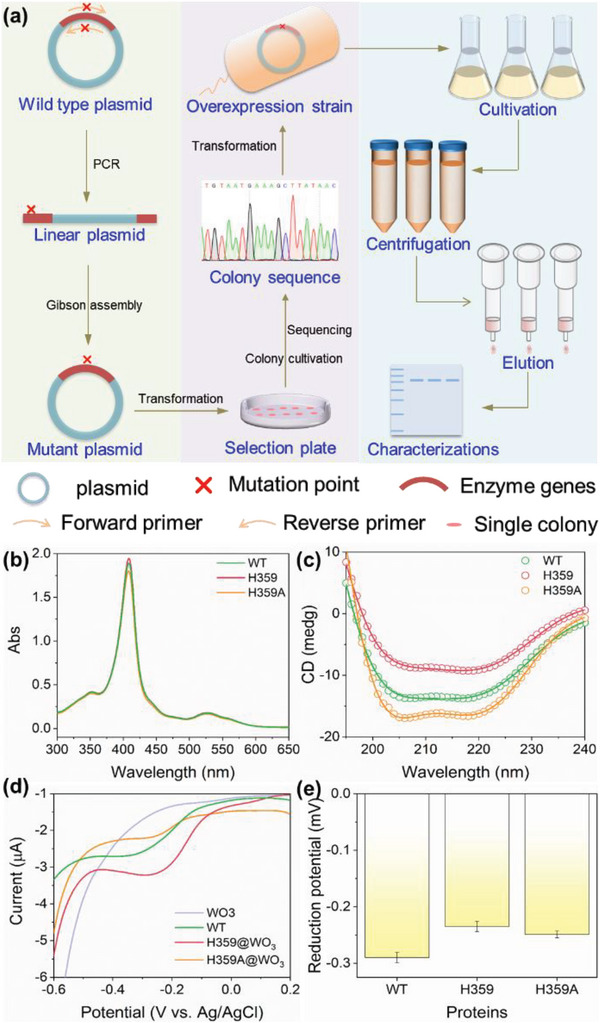
Purification and characterization of OmcA and its variants. a) The process to purify various proteins. b) UV–vis spectra of the three proteins. c) CD comparison of the three proteins. d) Electrochemical properties of WT, H359 and H359A on the h‐WO_3_ modified electrode. e) Redox potentials of the three proteins measured at least three times.

To validate the simulation results, electrochemical investigations were carried out to study the electron transfer from OmcA and its variants to h‐WO_3_. Strains containing the overexpression plasmids of the two variants were obtained by site‐directed mutagenesis (**Figure** **4**a), and the primers for PCR are listed in Table S4 (Supporting Information). The base sequences of the acquired plasmids were confirmed to be the same as designed by measuring with forward primer 5′‐GCGAATGCGCATTTCGATTGG‐3′ (Figure S5a, Supporting Information). Moreover, a comparison of DNA sequences and amino acid sequences of the three proteins, including WT, shows good alignment with each other (Figure S5b, Supporting Information). These results confirm that the overexpression strains were successfully constructed.

Using these strains, proteins were purified following the workflow (Figure 4a) and characterized immediately. As shown in SDS‐PAGE (Figure S6a, Supporting Information), a single band, located between 70‐ and 100‐kDa, was observed in each lane, indicating that the three proteins were of high purity. Comparing the structure of WT with those of the two variants predicted by Swiss Model, the overall structures of the three proteins matched well with each other (Figure S6b, Supporting Information), indicating that their structures were almost identical. This conclusion was further supported by their similar UV–vis and CD spectra (Figure 4b,c). Upon magnifying the mutation site, the different coordination states of heme 5 were observed. Specifically, WT OmcA maintained coordination with an axial His, while the axial ligand was absent for H359A, as Ala does not have an imidazole ring (Figure S6c, Supporting Information). For the H359 variant, there was a His 358 near heme 5. However, the distance between N atom of His 358 and the iron atom of heme 5 in H359 was longer (≈4.43 Å) that that in WT (1.96 Å). Therefore, His 358 did not coordinate with iron, exhibiting a cavity at one side of porphyrin ring, which provided a similar coordination environment as H359A.

To further explore the interfacial electron transfer catalytic process, the h‐WO_3_ nanorods were synthesized and prepared as the working electrode for electrochemical characterizations. The XRD pattern of the synthesized WO_3_ was the same as that in our previous report (Figure S7a, Supporting Information),^[^ ^24^ ^]^ which was further indexed to the h‐WO_3_ (JCPDS 85–2460). The sharp peaks demonstrate that the obtained WO_3_ was well‐crystallized. Additionally, the samples were uniform nanorods (Figure S7b, Supporting Information). These results indicate that the prepared h‐WO_3_ was appropriate to prepare electrodes for electrochemical analysis. Electrochemical behaviors of the active center (hemin) in OmcA and its variants were first evaluated on the h‐WO_3_ modified glassy carbon (GC) electrode. The cyclic voltammetries (CVs) of the hemin/h‐WO_3_/GC, h‐WO_3_/GC, hemin/GC and GC electrodes in minimal salts medium were recorded at 100 mV s^−1^ (Figure S8a, Supporting Information). No peaks were observed at either the bare or the h‐WO_3_/GC electrode. However, a couple of stable and well‐defined peaks were observed at the hemin/h‐WO_3_/GC electrode, and the more pronounced cathodic peak was attributed to the absorbed hemin with limited movement and concertation on the electrode surface. Hemin directly adsorbed on the GC electrode displayed a pair of small peaks at almost similar levels of potential. These results confirm that the interaction between the h‐WO_3_ and hemin improved both the electronic conductivity and electrochemical activity of hemin. The reduction peak potential was about −0.4 V (vs. Ag/AgCl), i.e., −0.2 V (vs. NHE), which is consistent with the value of OM *c*‐Cyt (−0.32–−0.1 V vs. NHE).^[^ ^22^ ^]^ Thus, the redox active center, heme, played an important role in EET from EAB to h‐WO_3_ via OM *c*‐Cyt. Figure S8b (Supporting Information) shows the CVs of hemin on the h‐WO_3_ in minimal salts medium at variable scan rates. Both the anodic and cathodic peak currents (*I*_pa_ and *I*_pc_) varied linearly with the potential scan rate (*v*) in the range of 10–250 mV s^−1^ (Figure S8c, Supporting Information), indicating a surface‐controlled process for the redox behavior of hemin on the h‐WO_3_. Thus, the h‐WO_3_ nanostructure could provide an appropriate microenvironment to host the redox active center of *c*‐Cyts and maintain its bioactivity.

After giving insights into the redox ability of the active center, differential pulse voltammetry was conducted for the proteins on the h‐WO_3_ modified pyrolytic graphite electrodes. Compared to the WT, the redox potentials of H359 and H359A shifted positively (Figure 4d,e), indicating a higher driving force (potential difference) for the electron transfer from two variants to the h‐WO_3_. Thus, the electron transfer from the two variants to the h‐WO_3_, the reverse reaction of protein reduction, required more energy in comparison with the WT. In other words, the WT OmcA with bis‐His as axial ligands was more beneficial for the h‐WO_3_ to capture electrons, implying that the axial His exerted an impact on electron flow from cytochrome to h‐WO_3_. With α‐Fe_2_O_3_ as the electron acceptor, OmcA can bind to the hematite surface via the binding motif that is covalently connected to the axial His ligand of heme.^[^ ^25^ ^]^ This result suggests the important role of the axial His in the ET of OmcA to α‐Fe_2_O_3_. When delivering electrons for the conversion of H_2_O_2_ to H_2_O, the axial His ligation of heme in MtrC became loosely bound, thus distorting the heme plane or stabilizing the intermediate by binding the electron acceptor.^[^ ^10^ ^]^ In OmcZ nanowires, the axial His ligands lock T‐stacked hemes tightly together, resulting in a close distance between T‐stacked hemes for electron transfer.^[^ ^8b^ ^]^ Owing to the importance of the axial ligand in regulating the redox potentials of cytochromes,^[^ ^26^ ^]^ the axial His ligand with electron donating nature exerts a great impact on the ET process of soluble periplasmic cytochromes.^[^ ^27^ ^]^ Beyond these *c*‐Cyts, the axial cysteine ligation of cytochromes P450 can facilitate the O─O bond heterolysis,^[^ ^28^ ^]^ indicating the significance of the axial ligand for the catalytic reaction. Moreover, the replacement of axial cysteine with His ligation induces an orientation change of the porphyrin ring in cytochrome P450 from *Sulfolobus acidocaldarius*.^[^ ^29^ ^]^ These results indicate the importance of the axial His ligation in ET process and the universality of redox catalysis in cytochromes.

**Figure 5:**
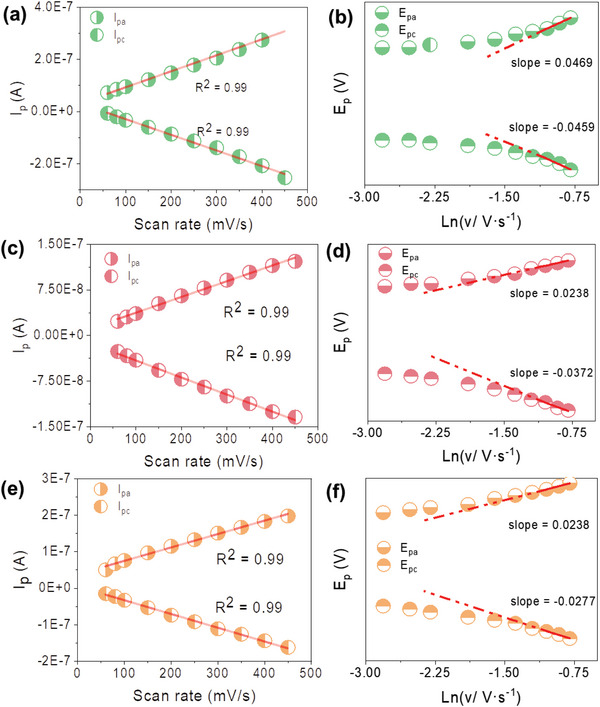
Correlations of the peak current versus the scan rate and relationships between the redox peaks and the natural logarithm of scan rate. Linear correlations between the peak currents of the a) WT, c) H359, and e) H359A with the scan rates. Correlations of the redox potentials of the b) WT, d) H359, and f) H359A with the natural logarithm of the scan rate (ln (*v*)).

To estimate the interfacial electron transfer rate, CVs under various scan rates were further conducted. Owing to the weak redox peaks of *c*‐Cyts on the h‐WO_3_ modified electrodes (Figure S9, Supporting Information), Au electrodes with good biocompatibility, excellent stability, and special electrical properties were selected as the working electrodes.^[^ ^30^ ^]^ The three proteins exhibited visible oxidation and reduction peaks (Figure S10, Supporting Information). Moreover, the redox potentials shifted positively after deleting His 359 or replacing it with alanine, being consistent with the results in Figure 4d. Therefore, the interfacial electron transfer rates were estimated by conducting CVs with various scan rates. For all the three proteins, the peak current increased linearly with the increasing scan rate (**Figure** **5**), indicating a surface‐controlled electrochemical process. Thus, the correlation of the redox potentials with the natural logarithm of scan rate was further analyzed based on Laviron theory.^[^ ^13^ ^]^ According to the plots, the electron‐transfer coefficient (α_s_) was estimated to be 0.505, 0.390, and 0.462 for the WT, H359 and H359A, respectively. Assuming it is a one‐electron transfer reaction, the interfacial electron‐transfer rate constant (*k*_s_) was estimated to be 0.505, 0.390, and 0.462 s^−1^ for the WT, H359 and H359A, respectively. Moreover, the estimated rate constants were in accord with the reported electron transfer rate (≈0.1 s^−1^/cytochrome) between OmcA and Fe(III) nanoparticles measured by fluorescence correlation spectroscopy.^[^ ^31^ ^]^ These experiments were conducted twice independently, again, the WT OmcA outperformed its two variants in interfacial electron transfer. These results indicate that the WT OmcA with His ligand exhibits the fastest electron transfer rate. Thus, the site‐directed mutagenesis offers an effective approach to tune the abiotic/biotic interfacial electron transfer reaction.

### 2.5 Energy Transfer in OmcA/h‐WO_3_ Systems

**Figure 6:**
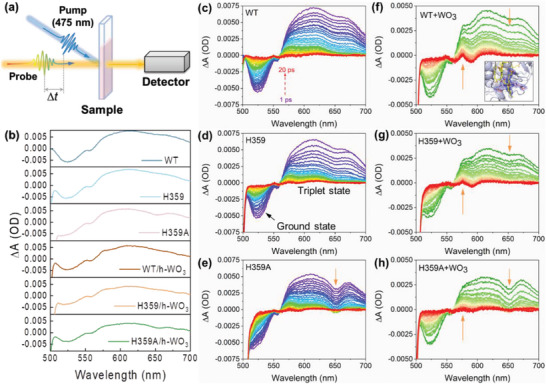
TAS of the excited states of OmcA and its variants on h‐WO_3_ surface. a) The working diagram of TAS. b) TAS of the proteins at 1 ps under various conditions. Time‐dependent TAS of c) WT, d) H359 , e) H359A, f) WT/h‐WO_3_, g) H359/h‐WO_3_, and h) H359A/h‐WO_3_.

The EET process allows microbes to gain energy by respiring on extracellular solid minerals.^[^ ^32^ ^]^ TAS was adopted to further explore the energy transfer at the interface of proteins and h‐WO_3_ (**Figure** **6**). The spectra of the three proteins, including the ground state peak at ≈525 nm and the broad excited state absorption from 575 to 700 nm, were similar to each other (Figure 6c–e), indicating that the overall structure of OmcA was maintained after the mutation at His 359 site. This result is consistent with the electrochemical and spectroscopic characterizations shown in Figure 4b,c. For H359A, the peak shape of the triplet state was different from those of WT and H359, suggesting the existence of special structural information in peptides (Figure 6e,h). Notably, the lifetime of triplet state of the WT OmcA was extended when the solid electron acceptor was dosed into the system. The signal (≈575 nm) in the WT/h‐WO_3_ remained after 20 ps (Figure 6f) but disappeared in the H359A/h‐WO_3_ (Figure 6h), suggesting that the energy transfer could also be adjusted by the axial coordinated His residue. The lifetime difference of the triplet state might be attributed to the different degrees of conjugation around the terminal electron flow site, heme 5.^[^ ^33^ ^]^ The electron density map of the π‐conjugated bis‐His‐Heme 5 in WT shows a continuous chain from His 359 to 368 (Figure S11, Supporting Information). The conjugation degree around heme 5 in H359 and H359A was lower than that in WT, thus reducing the triplet lifetime.

The results of this work highlight that the interfacial electron transfer from OmcA to the h‐WO_3_ nanomaterials is influenced by the microstructure features of the cytochrome, the dynamic properties of the system, the orientation of porphyrin ring, and the interaction between the active center and the crystal surface of h‐WO_3_. The electrochemical results confirm the h‐WO_3_ can improve the electronic conductivity and the electrochemical activity of the redox active center of OM *c*‐Cyts. Moreover, the excellent performance of a microbial fuel cell inoculated with *S. oneidensis* MR‐1 using h‐WO_3_ nanorods as an electrode material demonstrates a stable EET process from microbes to the h‐WO_3_ nanorods.^[^ ^24^ ^]^ These results imply that the rational design and preparation of electron‐conducting proteins to facilitate efficient electron transfer can provide a direction for exploring electron transfer at biotic/abiotic interfaces.

## 3 Conclusion

In this work, an integrated approach via coupling theoretical calculations with electrochemical and spectroscopy experiments is adopted to elucidate the mechanism of electron transfer catalysis at the protein/nanomaterial interface. The electron transfer rate at the interface is found to be dependent primarily on the microenvironment of the solvent‐exposed active center of the OM *c*‐Cyts. Microscopic distributions reflected by RDFs reveal a distance of ≈6.7 Å for the first shell of the heme near the crystal surface. Kinetic analysis demonstrates that the axial coordinated His significantly impacts the electron transfer rate. In addition, the interfacial electron transfer can occur with a thermodynamic driving force. The conformation of porphyrin ring with respect to the h‐WO_3_ nanocluster surface is crucial for lowering the *E*_a_, enabling electron transfer at the interface to occurs at a high constant rate and resulting in rapid reaction equilibrium. The electron transfer efficiency is governed by the special molecular configuration of cytochrome, and the apparent electron transfer rate can be regulated by the site‐directed mutagenesis. The results of our work lay a foundation for applying h‐WO_3_ as a platform for exploring the functions of genes related to the microbial EET. Furthermore, our approach facilitates a better understanding about the importance of the microbe‐nanostructure interactions that are required for designing highly‐efficient biotic‐linked‐abiotic bioelectrocatalytic systems.

## 4 Experimental Section

### DFT Calculations

The geometric optimization and the calculations of thermodynamic and kinetic properties for interfacial electron transfer were carried out by DFT calculations. The WO_3_ cluster was terminated with hydrogen atoms. The heme models with different axial ligands originated from OmcA were constructed. In the calculations, the Perdew–Bruke–Ernzerhof (PBE) functional of the general gradient approximation (GGA) including *p* polarization (DNP) and all‐electron relativistic effects were adopted in DMol^3^ package.^[^ ^34^ ^]^ To reflect the aqueous solution environment, the conductor‐like screening model (COSMO) was adopted. Spin polarization had been considered and Tkatchenko–Scheffler (TS) method was used to correct the non‐bonding effects in the calculation process. The convergence criteria of energy, maximum force and maximum displacement were set to 1 × 10^−5^ hartree, 0.002 hartree Å^−1^ and 0.005 Å, respectively. Moreover, the smearing was applied to the orbital occupation to speed up convergence with the value of 0.02 hartree. To obtain energy barriers along the minimum energy reaction pathways, complete LST/QST method was used with RMS convergence of 0.01 hartree Å^−1^.

### MD Simulations

The code for the OmcA (WT) of *S. oneidensis* MR‐1 was 4LMH in the protein data bank. The variants (H359, H359A) were built from SWISS‐MODEL.^[^ ^35^ ^]^ The model contained the OmcA or its variants in aqueous solution on the (001) crystal surface of h‐WO_3_ nanocluster. The MD boxes of the WT/h‐WO_3_, H359/h‐WO_3_, and H359A/h‐WO_3_ in aqueous solution were first calculated with the energy minimization step (details in Supporting Information), then relaxed in NVT ensemble for 5 ns at 298.15 K. The models were equilibrated by using a time step of 1.0 fs, the Andersen algorithm with a collision ratio of 1.0, and the Berendsen algorithm with a decay constant of 0.1 ps. The force field of Universal was adopted.

### Site‐Directed Mutagenesis and Protein Purification

Since His 359 was axially coordinated to heme 5 that functions as the electron outlet in *Shewanella*, this amino acid was chosen to construct OmcA mutations. Specifically, plasmids for H359A and His359 deletion were constructed by site‐directed mutagenesis using the wild type OmcA (pBAD) as the template for the polymerase chain reaction (PCR) procedure. The primers were synthesized by Sangon Co., China and were listed in Table S4 (Supporting Information). The sequences of the plasmids were measured with forward primer 5′‐GCGAATGCGCATTTCGATTGG‐3′. Then, these plasmids were introduced into *S. oneidensis* MR‐1 deficient in expressing OmcA and MtrC by electroporation strategy. Afterward, OmcA and mutant OmcA were expressed and purified as before. Briefly, the overexpression strains were cultivated at 30 °C in LB media containing 30 mg L^−1^ kanamycin and 1 mm L‐arabinose was added when the cells reached the log phase. After growing overnight, the supernatant containing the proteins were collected via centrifugation at 8000 g for 30 min and incubated with pre‐equilibrated nickel‐charged Hitrap NTA beads overnight at 4 °C. The beads absorbing proteins were packed for protein elution. Specifically, 20 mm HEPES buffer (pH 7.8) containing 10 mm imidazole and 10% glycerol was used to remove non‐specific binding protein. Then, OmcA was eluted with 250 mm imidazole followed by sodium dodecyl sulfate polyacrylamide gel electrophoresis (SDS‐PAGE) characterization. After that, the protein was buffered with imidazole‐free system for storage at −80 °C.

### Electrochemical Experiments

The CV experiments were carried out on a CHI 660 electrochemical workstation (CHI Instruments Co., China) with a three‐electrode system. A glassy carbon (GC) electrode, a platinum wire, and an Ag/AgCl electrode were used as the working electrode, counter electrode, and reference electrode, respectively. The GC electrode (*d* = 3 mm) was polished with 0.3 and 0.05 µm alumina powder in succession, and then thoroughly sonicated in water and ethanol for a few minutes, respectively. The GC electrode was coated by casting 5 µL of h‐WO_3_ (0.5 mg mL^−1^) suspended in 10 mm Hepes and dried at room‐temperature without light. Crystalline h‐WO_3_ nanocluster were synthesized using a hydrothermal process with Na_2_WO_4_·2H_2_O as a precursor, as described inthe previous work.^[^ ^15a^ ^]^ Then, the modified electrode was immersed in 0.2 mg mL^−1^ hemin solution, which was dissolved in DMSO and diluted by 10 mm Hepes. After 30 min, the electrode was rinsed with deionized water softly and also dried at ambient temperature in a light resistant container. The CV tests were conducted in minimal salts medium at room temperature to reflect the electrochemical behavior of the redox active center of *c*‐Cyts in the EAB growth environment. Before the tests, the electrolyte was deoxygenated by purging nitrogen. All solutions were prepared from reagent‐grade chemicals without further purification. To estimate the interfacial electron transfer rate (*k*_s_), the *c*‐Cyts modified Au electrodes were selected as the working electrodes and the scan rates of CV were ranging from 60 to 450 mV s^−1^. Specifically, the Au electrodes were activated in 0.5 m H_2_SO_4_ by CV with a scan rate of 100 mV s^−1^ after being polished and sonicated. The activated Au electrodes were immersed in *c*‐Cyts solution overnight for surface modification. To analyse the electron transfer between OmcA or its variants and h‐WO_3_, concentrated proteins were cast on h‐WO_3_ modified pyrolytic graphite electrodes and functioned as the working electrode. The differential pulse voltammetry was conducted at least three times in degassed buffer with potential range from 0.2 to −0.6 V. The other parameters are listed below: increment was 5 mV, amplitude was 50 mV, pulse width was 50 ms, sample width was 20 ms and the pulse period was 500 ms.

### Transient Absorption Spectroscopy

A pump‐probe system (Helios, Ultrafast System LLC Co., USA) coupled with an amplified femtosecond laser system (Coherent) was utilized to collect TAS under ambient conditions. To avoid excitation of h‐WO_3_, a 475 nm pump pulse (≈40 nJ pulse^−1^ at the sample) was selected and delivered by an optical parametric amplifier (TOPAS‐800‐fs). The amplifier was excited by a Ti:Sapphire regenerative amplifier and the detailed parameters were as follow: pulse duration 35 fs, center wavelength 800 nm, pulse energy 3 mJ, repetition rate 1 kHz. The white‐light continuum probe pulses were generated by focusing the 800 nm fs‐laser on a sapphire plate. A time delay of within 30 ps between the pump and probe pulses were maintained by a motorized optical delay line. The probe light was directed to samples in a 2 mm quartz cuvette.

## Conflict of Interest

The authors declare no conflict of interest.

## Author Contributions

S.S.Y. and X.Y.Z. contributed equally to this work. S.S.Y., S.J.Y., J.J.C. and H.Q.Y. designed the research; S.S.Y. performed the experimental section; X.Y.Z. conducted the theoretical calculations; S.S.Y., X.Y.Z., S. L. J., Q.Z., and J.J.C. analyzed the data; S.S.Y., X.Y.Z., J.J.C., and H.Q.Y. wrote the manuscript.

## Supporting information

Supporting Information

## Data Availability

The data that support the findings of this study are available from the corresponding author upon reasonable request.

## References

[1] J. Zhao , F. Li , S. Kong , T. Chen , H. Song , Z. Wang , Adv. Sci. 2023, 10, 2206622. 10.1002/advs.202206622 PMC10037984 36710254

[2] a) X. Shao , Z. Ding , W. Zhou , Y. Li , Z. Li , H. Cui , X. Lin , G. Cao , B. Cheng , H. Sun , M. Li , K. Liu , D. Lu , S. Geng , W. Shi , G. Zhang , Q. Song , L. Chen , G. Wang , W. Su , L. Cai , L. Fang , D. T. Leong , Y. Li , X. F. Yu , H. Li , Nat. Nanotechnol. 2021, 16, 1150; 34354264 10.1038/s41565-021-00952-x

[3] C. Kaspar , B. J. Ravoo , W. G. van der Wiel , S. V. Wegner , W. H. P. Pernice , Nature 2021, 594, 345. 34135518 10.1038/s41586-021-03453-y

[4] L. Shi , H. Dong , G. Reguera , H. Beyenal , A. Lu , J. Liu , H. Q. Yu , J. K. Fredrickson , Nat. Rev. Microbiol. 2016, 14, 651. 27573579 10.1038/nrmicro.2016.93

[5] a) Z. Guo , J. J. Richardson , B. Kong , K. Liang , Sci. Adv. 2020, 6, eaaz0330; 32206719 10.1126/sciadv.aaz0330 PMC7080450

[6] a) D. R. Lovley , J. F. Stolz , G. L. Nord , E. J. P. Phillips , Nature 1987, 330, 252;

[7] T. Fukushima , S. Gupta , B. Rad , J. A. Cornejo , C. J. Petzold , L. J. G. Chan , R. A. Mizrahi , C. Y. Ralston , C. M. Ajo‐Franklin , J. Am. Chem. Soc. 2017, 139, 12647. 28806874 10.1021/jacs.7b06560

[8] a) Y. Jiao , Y. Qiu , L. Zhang , W. G. Liu , H. Mao , H. Chen , Y. Feng , K. Cai , D. Shen , B. Song , X. Y. Chen , X. Li , X. Zhao , R. M. Young , C. L. Stern , M. R. Wasielewski , R. D. Astumian , W. A. Goddard, 3rd , J. F. Stoddart , Nature 2022, 603, 265; 35264758 10.1038/s41586-021-04377-3

[9] T. T. Zhu , Z. H. Cheng , S. S. Yu , W. W. Li , D. F. Liu , H. Q. Yu , Environ. Microbiol. 2022, 24, 1838. 35170205 10.1111/1462-2920.15939

[10] B. Reuillard , K. H. Ly , P. Hildebrandt , L. J. Jeuken , J. N. Butt , E. Reisner , J. Am. Chem. Soc. 2017, 139, 3324. 28221032 10.1021/jacs.6b12437 PMC5411108

[11] F. Wang , Y. Gu , J. P. O'Brien , S. M. Yi , S. E. Yalcin , V. Srikanth , C. Shen , D. Vu , N. L. Ing , A. I. Hochbaum , E. H. Egelman , N. S. Malvankar , Cell 2019, 177, 361. 30951668 10.1016/j.cell.2019.03.029 PMC6720112

[12] a) M. J. Edwards , N. A. Baiden , A. Johs , S. J. Tomanicek , L. Liang , L. Shi , J. K. Fredrickson , J. M. Zachara , A. J. Gates , J. N. Butt , D. J. Richardson , T. A. Clarke , FEBS Lett. 2014, 588, 1886; 24747425 10.1016/j.febslet.2014.04.013

[13] E. Laviron , J. Electroanal. Chem. 1974, 52, 355.

[14] a) A. C. Mitchell , L. Peterson , C. L. Reardon , S. B. Reed , D. E. Culley , M. R. Romine , G. G. Geesey , Geobiology 2012, 10, 355; 22360295 10.1111/j.1472-4669.2012.00321.x

[15] a) S. J. Yuan , W. W. Li , Y. Y. Cheng , H. He , J. J. Chen , Z. H. Tong , Z. Q. Lin , F. Zhang , G. P. Sheng , H. Q. Yu , Nat. Protoc. 2014, 9, 112; 24356770 10.1038/nprot.2013.173

[16] J.‐H. Wu , T. Tian , Y.‐F. Guan , F. Zhang , H.‐Q. Yu , Environ. Sci: Nano 2022, 9, 2764.

[17] B. W. Faughnan , R. S. Crandall , P. M. Hyman , RCA Rev 1975, 36, 177.

[18] O. Einsle , A. Messerschmidt , P. Stach , G. P. Bourenkov , H. D. Bartunik , R. Huber , P. M. Kroneck , Nature 1999, 400, 476. 10440380 10.1038/22802

[19] N. L. Costa , T. A. Clarke , L. A. Philipp , J. Gescher , R. O. Louro , C. M. Paquete , Bioresour. Technol. 2018, 255, 308. 29444758 10.1016/j.biortech.2018.01.133

[20] X. W. Liu , Y. X. Huang , X. F. Sun , G. P. Sheng , F. Zhao , S. G. Wang , H. Q. Yu , ACS Appl. Mater. Interfaces 2014, 6, 8158. 24818709 10.1021/am500624k

[21] S. H. Kopf , C. Henny , D. K. Newman , Environ. Sci. Technol. 2013, 47, 2602. 23402562 10.1021/es3049459 PMC3604861

[22] a) S. J. Field , P. S. Dobbin , M. R. Cheesman , N. J. Watmough , A. J. Thomson , D. J. Richardson , J. Biol. Chem. 2000, 275, 8515; 10722689 10.1074/jbc.275.12.8515

[23] a) Z. M. Summers , J. A. Gralnick , D. R. Bond , mBio 2013, 4, e00420; 10.1128/mBio.00420-12 PMC3560526 23362318

[24] F. Zhang , S.‐J. Yuan , W.‐W. Li , J.‐J. Chen , C.‐C. Ko , H.‐Q. Yu , Electrochim. Acta 2015, 152, 1.

[25] B. H. Lower , R. D. Lins , Z. Oestreicher , T. P. Straatsma , M. F. Hochella , L. A. Shi , S. K. Lower , Environ. Sci. Technol. 2008, 42, 3821. 18546729 10.1021/es702688c

[26] J. Liu , S. Chakraborty , P. Hosseinzadeh , Y. Yu , S. Tian , I. Petrik , A. Bhagi , Y. Lu , Chem. Rev. 2014, 114, 4366. 24758379 10.1021/cr400479b PMC4002152

[27] T. A. Clarke , M. J. Edwards , A. J. Gates , A. Hall , G. F. White , J. Bradley , C. L. Reardon , L. Shi , A. S. Beliaev , M. J. Marshall , Z. Wang , N. J. Watmough , J. K. Fredrickson , J. M. Zachara , J. N. Butt , D. J. Richardson , Proc. Natl. Acad. Sci. U.S.A. 2011, 108, 9384. 21606337 10.1073/pnas.1017200108 PMC3111324

[28] S. Sivaramakrishnan , H. Ouellet , H. Matsumura , S. Guan , P. Moenne‐Loccoz , A. L. Burlingame , P. R. O. de Montellano , J. Am. Chem. Soc. 2012, 134, 6673. 22444582 10.1021/ja211499q PMC3329582

[29] J. A. McIntosh , T. Heel , A. R. Buller , L. Chio , F. H. Arnold , J. Am. Chem. Soc. 2015, 137, 13861. 26299431 10.1021/jacs.5b07107 PMC4635421

[30] M. Kizling , M. Dzwonek , A. Wieckowska , R. Bilewicz , Curr. Opin. Electrochem. 2018, 12, 113.

[31] L. S. Yijia Xiong , B. Chen , M. Uljana Mayer , B. H. Lower , Y. Londer , S. Bose , M. F. Hochella , J. K. Fredrickson , T. C. Squier , J. Am. Chem. Soc. 2006, 128, 13978. 17061851 10.1021/ja063526d

[32] S. Mishra , S. Pirbadian , A. K. Mondal , M. Y. El‐Naggar , R. Naaman , J. Am. Chem. Soc. 2019, 141, 19198. 31702906 10.1021/jacs.9b09262

[33] L.‐S. Cui , A. J. Gillett , S.‐F. Zhang , H. Ye , Y. Liu , X.‐K. Chen , Z.‐S. Lin , E. W. Evans , W. K. Myers , T. K. Ronson , H. Nakanotani , S. Reineke , J.‐L. Bredas , C. Adachi , R. H. Friend , Nat. Photonics 2020, 14, 636.

[34] J. D. Gale , A. L. Rohl , Mol. Simul. 2003, 29, 291.

[35] A. Waterhouse , M. Bertoni , S. Bienert , G. Studer , G. Tauriello , R. Gumienny , F. T. Heer , T. A. P. de Beer , C. Rempfer , L. Bordoli , R. Lepore , T. Schwede , Nucleic Acids Res. 2018, 46, W296. 29788355 10.1093/nar/gky427 PMC6030848

