# Supplementary material for: Electron Transfer Mechanism at the Interface of Multi‐Heme Cytochromes and Metal Oxide
Source: Adv Sci (Weinh). 2023 Aug 16;10(29):2302670. doi: 10.1002/advs.202302670 (PMC10582406; doi:10.1002/advs.202302670)
Supplement: Supplementary file 1 — Supporting Information [file ADVS-10-2302670-s001.pdf]

ADVANCED SCIENCE
Open Access

Supporting Information

for *Adv. Sci.*, DOI 10.1002/advs.202302670

Electron Transfer Mechanism at the Interface of Multi-Heme Cytochromes and Metal Oxide

*Sheng-Song Yu, Xin-Yu Zhang, Shi-Jie Yuan, Shen-Long Jiang, Qun Zhang, Jie-Jie Chen**
*and Han-Qing Yu**

Supporting Information

Electron Transfer Mechanism at the Interface of Multi-Heme Cytochromes and Metal Oxide

Sheng-Song Yu, Xin-Yu Zhang, Shi-Jie Yuan, Shen-Long Jiang, Qun Zhang, Jie-Jie Chen, Han-Qing Yu**

S.S. Yu, X.Y. Zhang, Prof. J.J. Chen, Prof. H.Q. Yu

Department of Environmental Science and Engineering, University of Science and Technology of China, Hefei 230026, China

S.J. Yuan

State Key Laboratory of Pollution Control and Resource Reuse, College of Environmental Science and Engineering, Tongji University, Shanghai 200092, China

S.L. Jiang, Q. Zhang

Department of Chemical Physics, University of Science and Technology of China, Hefei 230026, China

This supporting information is a 20-page document, including 4 tables, 11 figures, references, and this cover page.

NOMENCLATURE

EAB	electrochemically active bacteria
hemin	Porphyrin ring with Fe in oxidation state (Fe^{3+})
heme	Porphyrin ring with Fe in reduction state (Fe^{2+})
WT	OmcA in wide type
H359	OmcA with the His 359 removed
H359A	OmcA with the His 359 replaced by Ala
h-WO_3	hexagonal tungsten trioxide
OM	outer membrane
MD	molecular dynamics
His	histidine
RDF, g(r)	radial distribution function
EET	extracellular electron transfer
Bis-His-Heme	Heme axial coordinated with two His residues
Mono-His-Heme	Heme axial coordinated with one His residue
Ala-His-Heme	Heme axial coordinated with one His residue and with Ala on the other side of the porphyrin plane

Test S1. Energy Minimization Step

Models were calculated with the energy minimization step via the smart minimize method, which is a combination of the steepest descent, conjugated gradient, and Newton methods in a cascade. The convergence levels were set to 0.01, 0.01, and 0.1 kcal/(mol·Å), respectively. The nonbond interactions (i.e., electrostatic and van der Waals) were calculated by the Ewald summation method[1] with an accuracy of 0.01 kcal/mol. With the energy minimized models, molecular dynamics simulations were performed using a time step of 1.0 fs for equilibrating the electron transfer models and obtaining the final structures. All the molecular simulations were carried out using the Forcite module of Materials Studio package.

Test S2. Calculations of Gibb's Free Energy

The Gibb's free energy (ΔG_{ox}^{Θ}, Table S2) for the porphyrin oxidation under standard conditions are analyzed by DFT calculations. For all the electron transfer catalytic systems, the positive ΔG_{ox}^{Θ} values indicate that the free heme can not spontaneously transfer electrons to the nanocluster. Additionally, the experimental reduction potentials (E_{red}^{Θ}) of -0.32 to -0.10 V for outer membrane (OM) *c*-Cyt of *Shewanella vs.* normal hydrogen electrode (NHE)[2] *via* interaction with insoluble Fe(III) substrates indicate that the oxidation of *c*-Cyt is not thermodynamically spontaneous. This could be explained by E_{red}^{Θ} *vs.* NHE of the half-reaction:

$$E_{red}^{\Theta} = -\Delta G_{red}^{\Theta}/nF - E_{H}^{\Theta} \quad \text{(S1)}$$

where *n* is the number of electrons in the reaction, *F*, the Faraday constant, equals

23.06 kcal/mol V, and E_H^Θ is the standard reduction potential of NHE with a value of 4.28 V. Hence, when the E_{red}^Θ is larger than -4.28 V, the ΔG_{red}^Θ is negative.

The reported experimental E_{red}^Θ of OM cytochrome reveals that the ΔG_{red}^Θ is negative. Thus, the reverse reaction, the ΔG_{ox}^Θ is positive. The calculated results in Table S2 are in good agreement with the experimental results[2] and confirm that this model can be used to describe the electron transfer process from OM cytochromes by insoluble electron sinks.

References

[1] U. Essmann, L. Perera, M. L. Berkowitz, T. Darden, H. Lee, L. G. Pedersen, *J. Chem. Phys.* **1995**, *103*, 8577.

[2] a) S. J. Field, P. S. Dobbin, M. R. Cheesman, N. J. Watmough, A. J. Thomson, D. J. Richardson, *J. Biol. Chem.* **2000**, *275*, 8515; b) E. Marsili, D. B. Baron, I. D. Shikhare, D. Coursolle, J. A. Gralnick, D. R. Bond, *Proc. Natl. Acad. Sci. U.S.A.* **2008**, *105*, 3968.

Table S1. Geometry characteristic of porphyrin in the electron transfer catalytic process. Geometry comparison of the Fe coordinated ring in the optimized structures of different systems in the electron capturing process.

system	l(Fe-N_1) (Å)	l(Fe-N_2) (Å)	l(Fe-N_3) (Å)	l(Fe-N_4) (Å)	θ(N_1-Fe-N_3) (°)	θ(N_2-Fe-N_4) (°)
Bis-His-Heme(Ⅱ)/h-WO_3	2.001	1.987	1.993	1.995	178.498	179.025
Bis-His-Hemin(Ⅲ)/h-WO_3	1.995	1.997	2.013	1.974	178.282	178.966
Mono-His-Heme(Ⅱ)/h-WO_3	1.995	1.978	1.979	1.981	167.564	173.236
Mono-His-Hemin(Ⅲ)/h-WO_3	1.992	1.975	1.982	1.974	166.129	172.616
Ala-His-Heme(Ⅱ)/h-WO_3	1.989	1.984	1.975	1.991	168.099	169.355
Ala-His-Hemin(Ⅲ)/h-WO_3	1.998	1.980	1.982	1.980	165.246	171.926

Table S2. Energy change in the electron transfer catalysis between redox active center of cytochrome and h-WO_3. Energy change analysis of electron transfer for three systems, including the thermodynamic properties at ambient temperature (298.15 K) and atmosphere pressure (1 atm).

reaction	ΔG (eV)
Bis-His-Heme(II) ⇌ Bis-His-Hemin(III) + e^-	5.18
Mono-His-Heme(II) ⇌ Mono-His-Hemin(III) + e^-	5.20
Ala-His-Heme(II) ⇌ Ala-His-Hemin(III) + e^-	5.20

Table S3. Calculated electron transfer rate. Kinetic properties of electron transfer from porphyrin by h-WO_3 nanocluster, including energy barrier (E_a), standard entropy change of activation ($\Delta^{\neq}S^{\Theta}$) and rate constants (k_{et}).

reaction	E_a (eV)	$\Delta^{\neq}S^{\Theta}$ (eV/K)	k_{et} ($mol^{1-n}/L^{1-n}\cdot s$)
Bis-His-Heme(II) + $2H_2O$ ⇌ Bis-His-Hemin(III) + H_3O^+ + OH^- + e^-	3.37	0.04	1.09×10^{135}
Mono-His-Heme(II) + $2H_2O$ ⇌ Mono-His-Hemin(III) + H_3O^+ + OH^- + e^-	4.53	0.03	1.55×10^{102}
Ala-His-Heme(II) + $2H_2O$ ⇌ Ala-His-Hemin(III) + H_3O^+ + OH^- + e^-	4.69	0.03	7.08×10^{108}

Table S4. Primer sequences used in constructing and sequencing the mutants.

mutants	direction	sequence 5'→ 3'
H359	forward	GTCACACTGAAAAACCATCTGCTCACAGCAGCAC TGATTGTATGGCTTG
	reverse	CAAGCCATACAATCAGTGCTGCTGTGAGCAGATG GTTTTTCAGTGTGAC
H359A	forward	GTCACACTGAAAAACCATCTGCTCACGCAAGCAG CACTGATTGTATGGCTT
	reverse	AAGCCATACAATCAGTGCTGCTTGCGTGAGCAGA TGGTTTTTCAGTGTGAC

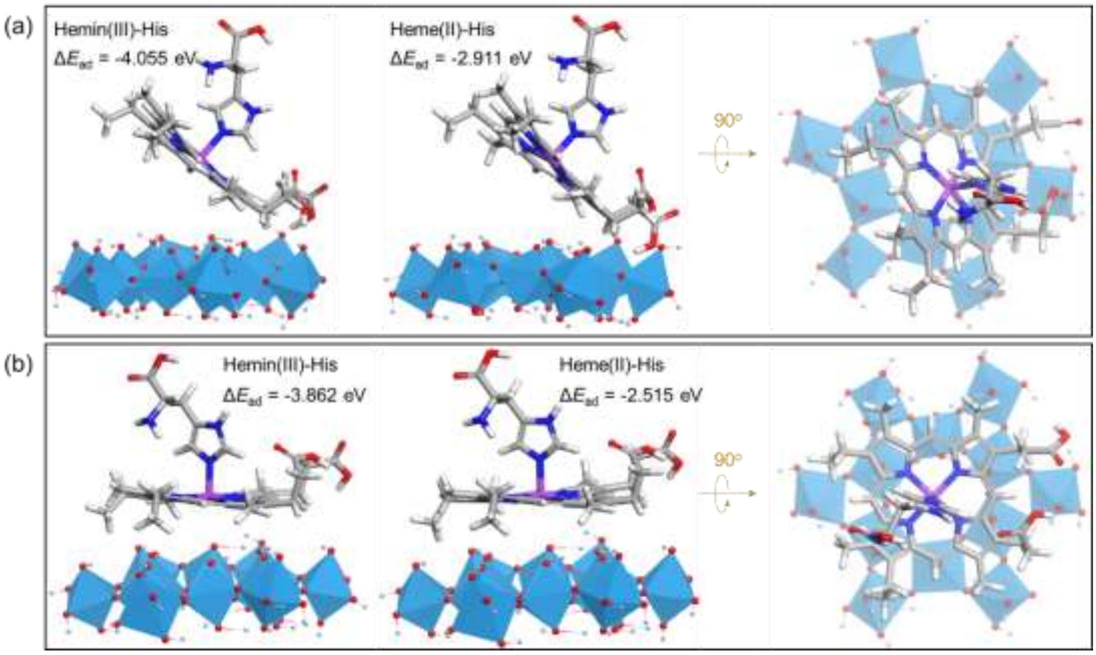

Figure S1. Optimized structures of the electrocatalytic systems with various orientations. (a) The electrocatalytic system of heme adopting a 45° orientation to the h-WO_3 surface. (b) The paralleled orientation of heme to the h-WO_3 surface.

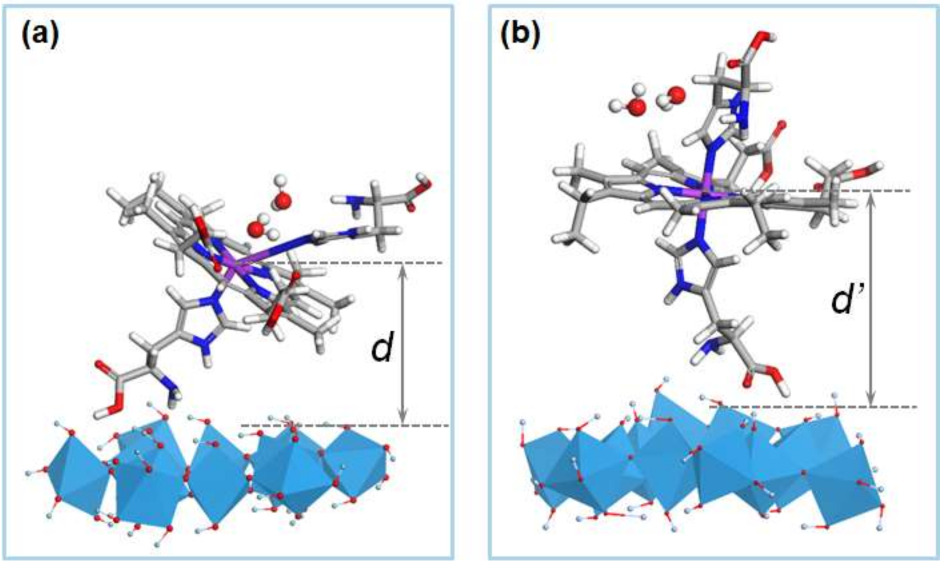

Figure S2. Orientations of bis-His-Heme on the h-WO_3 for electron transfer catalysis. (a) The distance between the heme and h-WO_3 in such an orientation can be reduced to accelerate the electron transfer. This orientation also presented in the equilibrium state after molecular dynamics simulations in the OmcA/h-WO_3 model. (b) This orientation of bis-His-Heme enlarges the distance of the electron transfer.

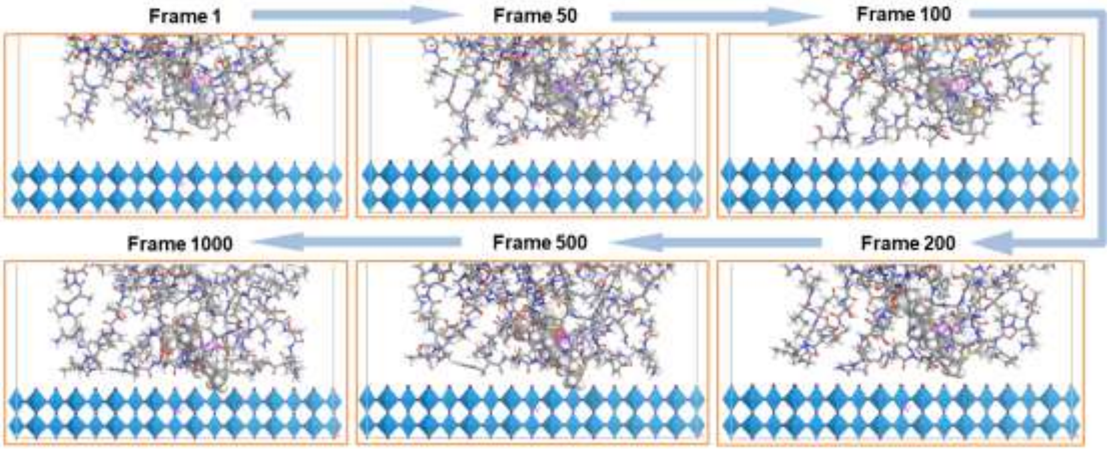

Figure S3. Relaxation of OmcA/h-WO_3 through molecular dynamics simulations. Relative position and configurations are presented in the selected frames. OmcA was gradually approaching to the surface of h-WO_3 through the simulations. All the H_2O molecules were deleted for clearly observing the configuration changes.

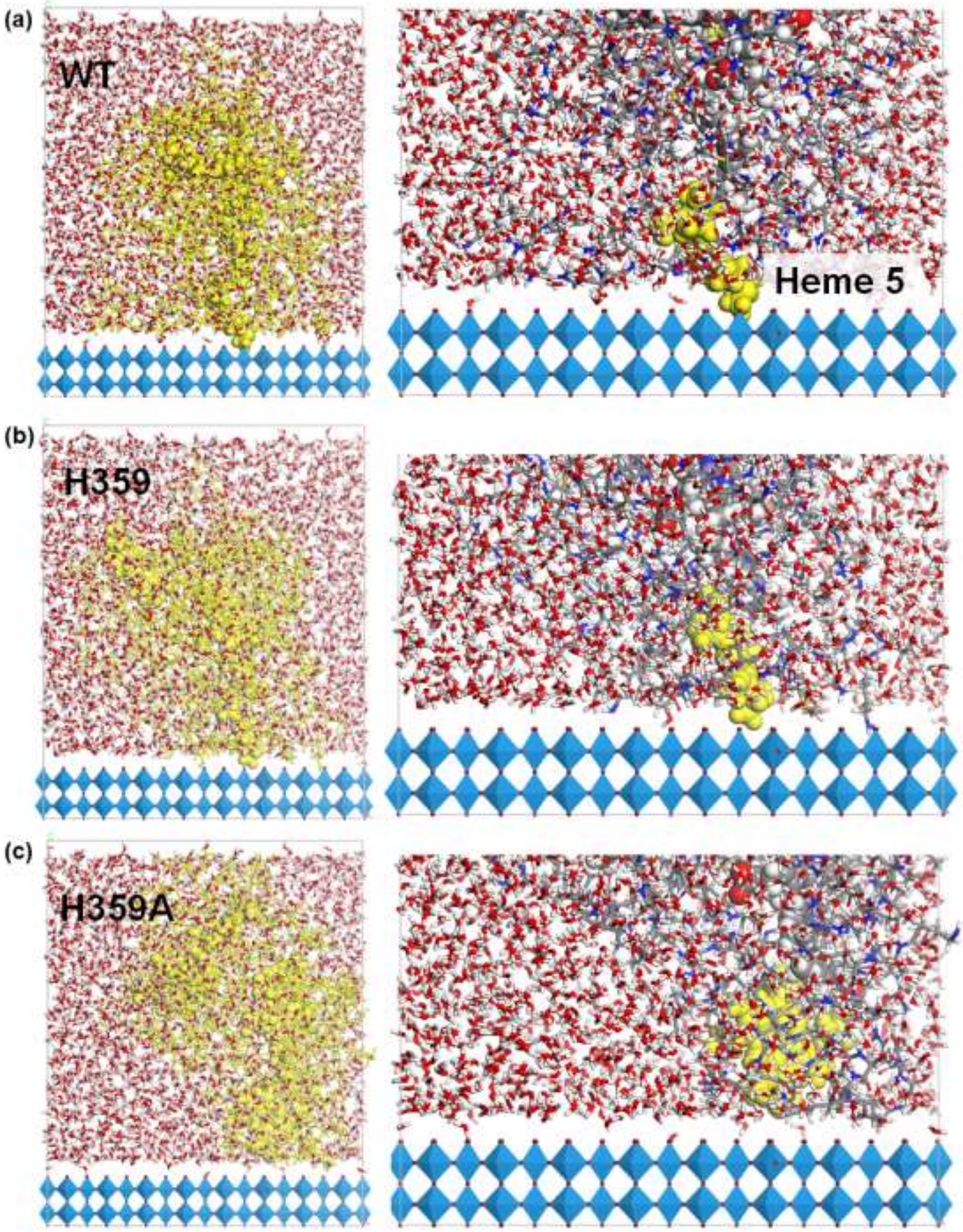

Figure S4. Snapshots of the solvated OmcA/h-WO_3 simulated system after equilibrium, WT (a), and the variants (b, c).

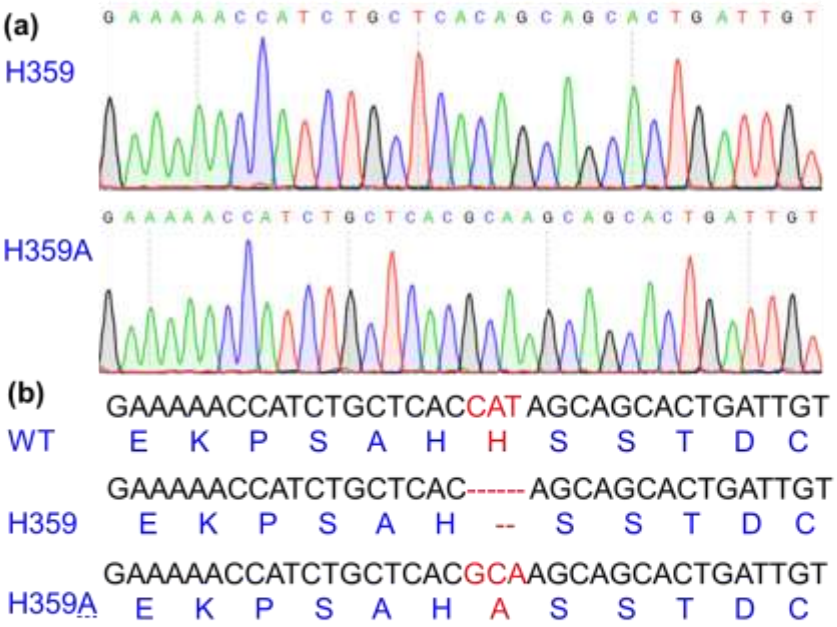

Figure S5. Comparison of DNA sequences and amino acid sequences of WT with H359 and H359A. (a) Sequencing results of the mutant plasmids with forward primer 5'-GCGAATGCGCATTTCGATTGG-3'. (b) Comparison of DNA and amino acid sequences among the three proteins.

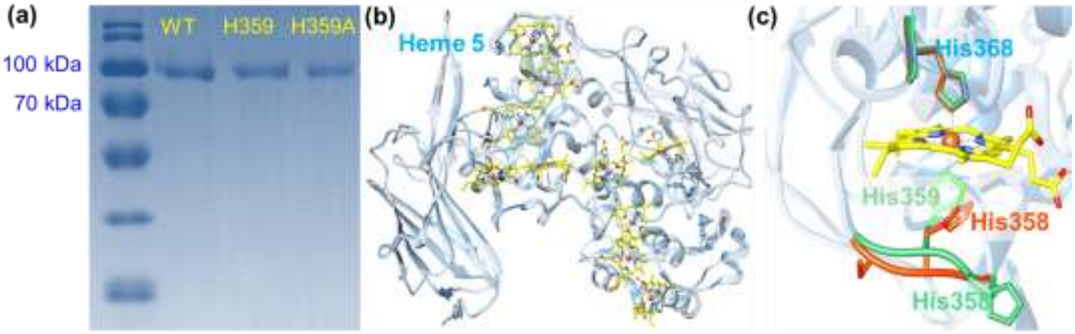

Figure S6. Structural comparison of WT, H359 and H359A. (a) SDS-PAGE of the three proteins. (b) Structural alignment of WT, H359 and H359A. (c) Coordination environments of heme 5 in various proteins. The axial ligands of heme 5 in WT and H359A are highlighted in green and cornflower blue, respectively. The overlapped residues exhibit dark green. The residues around heme 5 of H359 are highlighted in red.

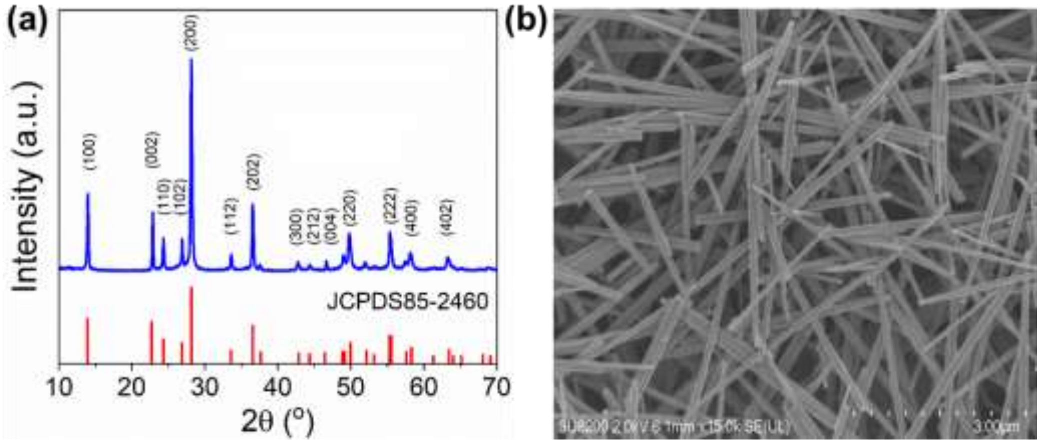

Figure S7. Characteristics of h-WO_3. XRD (a) and SEM (b) of synthesized h-WO_3.

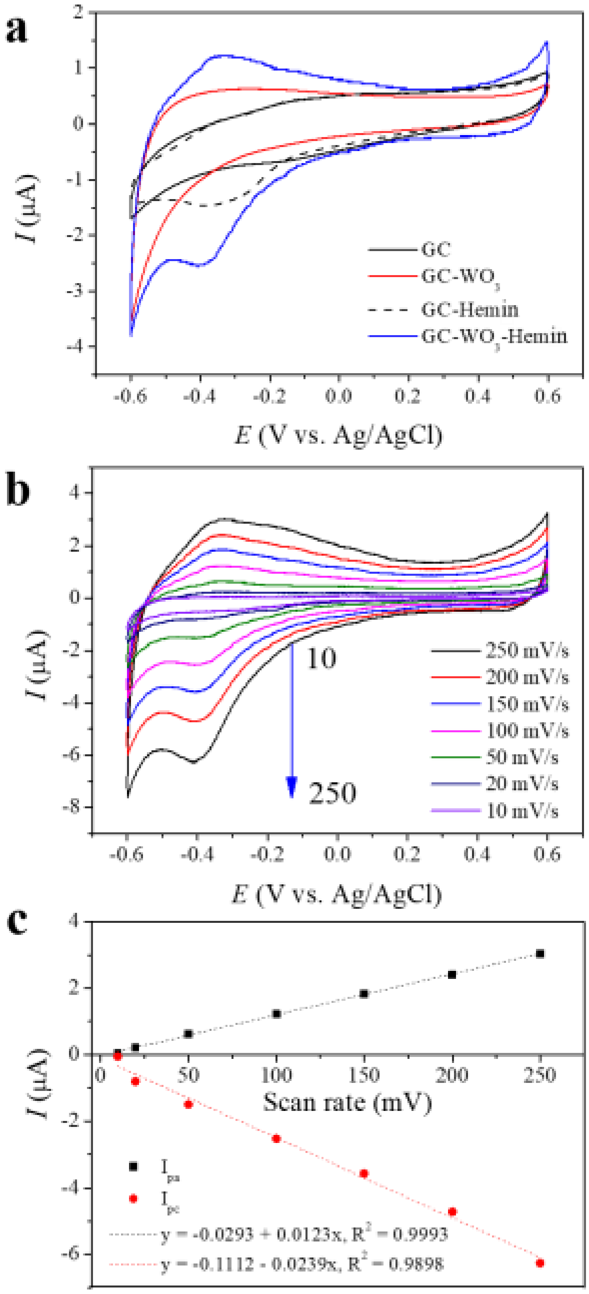

Figure S8. Electrochemical behaviors of hemin on h-WO_3. (a) CVs of hemin/h-WO_3/GC, h-WO_3/GC, hemin/GC and GC electrodes in minimal salts medium recorded at a scan rate of 100 mV/s. (b) CVs of hemin/h-WO_3/GC in minimal salts medium at various scan rates. (c) Plot of peak current *vs*. scan rate from 10 to 250 mV/s.

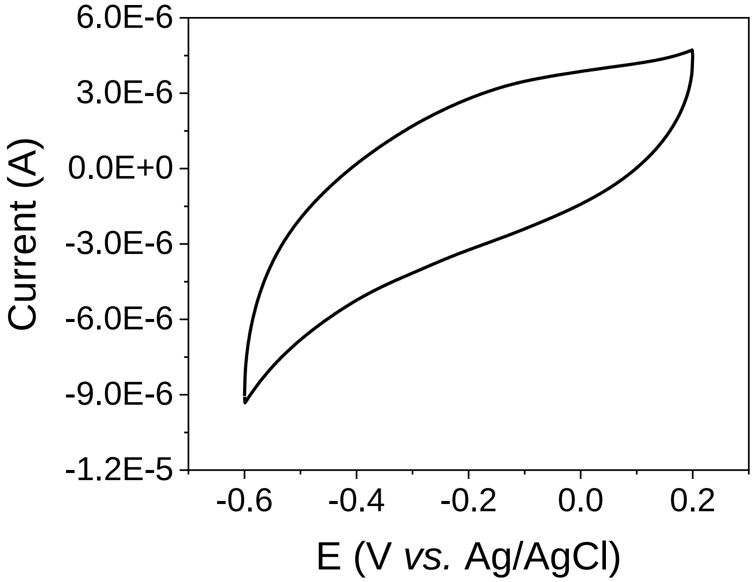

Figure S9. CV of WT OmcA on the pyrolytic graphite electrode modified with h-WO_3. Scan rate: 100 mV/s. Electrolyte: degassed 10 mM Hepes with 100 mM NaCl, pH 7.0.

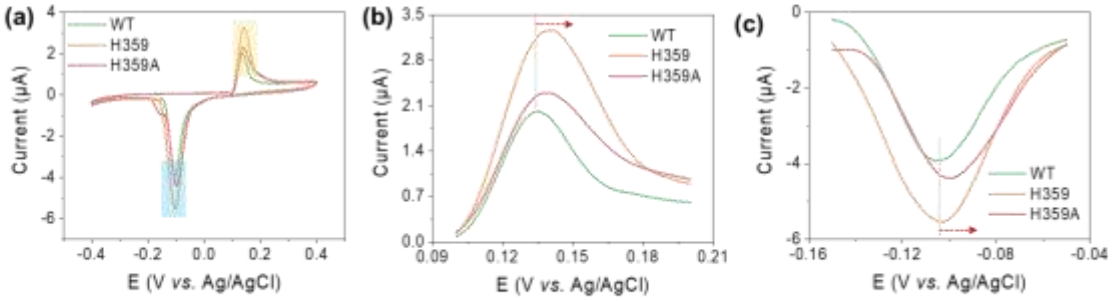

Figure S10. (a) CV of various types of OmcA on a gold electrode without h-WO_3. (b) The enlarged region of oxidation peaks. (c) The enlarged region of reduction peak. The red arrows indicate the positive shift of the redox potential after mutating.

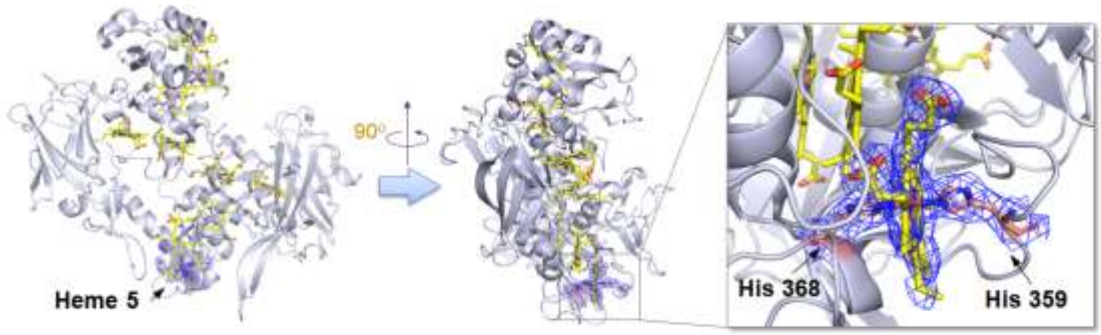

Figure S11. Electron density map of bis-His-Heme 5 in WT. The electron density map of π-conjugated bis-His-Heme shows a continuous chain from His 359 to 368.
